# Expanding Knowledge about the Influence of Citral on Cognitive Functions—In Vitro, In Vivo and Ex Vivo Studies

**DOI:** 10.3390/ijms25136866

**Published:** 2024-06-22

**Authors:** Karolina Wojtunik-Kulesza, Monika Rudkowska, Katarzyna Klimek, Monika Agacka-Mołdoch, Jarosław Mołdoch, Agnieszka Michalak

**Affiliations:** 1 Department of Inorganic Chemistry, Medical University of Lublin, Chodźki 4a, 20-093 Lublin, Poland; 2 Independent Laboratory of Behavioral Studies, Medical University of Lublin, 4A Chodźki, 20-093 Lublin, Poland; monika.rudkowska@umlub.pl (M.R.); agnieszka.michalak@umlub.pl (A.M.); 3 Department of Biochemistry and Biotechnology, Medical University of Lublin, 1 Chodźki Street, 20-093 Lublin, Poland; katarzyna.klimek@umlub.pl; 4 Department of Plant Breeding and Biotechnology, Institute of Soil Science and Plant Cultivation, State Research Institute, Czartoryskich 8, 24-100 Puławy, Poland; magacka@iung.pulawy.pl; 5 Department of Biochemistry and Crop Quality, Institute of Soil Science and Plant Cultivation, State Research Institute, Czartoryskich 8, 24-100 Puławy, Poland; jmoldoch@iung.pulawy.pl

**Keywords:** citral, memory, mice, brain, neurodegeneration, butyrylcholinesteraze, hepatotoxicity, passive avoidance test, locomotor activity, lipid peroxidation

## Abstract

Citral, a common monoterpene found in numerous plants, is an interesting compound that has been shown to have various biological activities. Although it is widely distributed in nature and there are many studies presenting its biological activities, its anti-neurodegenerative activity, especially under in vivo conditions, is very poorly understood. Thus, this paper aimed to deepen knowledge about citral activity towards factors and symptoms of neurodegeneration. To accomplish this, several comprehensive tests were conducted, including the estimation of butyrylcholinesterase inhibition, the evaluation of hepatotoxicity and the detection of oxidative stress and lipid peroxidation in vitro, as well as an in vivo behavioral assessment using mice models. Additionally, ex vivo determination of level of the compound in the brain and blood of a tested animal was undertaken. The results obtained revealed that citral is able to inhibit butyrylcholinesterase activity and protect hepatic cells against oxidative stress and lipid peroxidation in vitro. Moreover, behavioral tests in vivo indicated that citral (50 mg/kg) improves memory processes associated with acquisition (passive avoidance test), both in acute and subchronic administration. Additionally, we found that the administration of citral at 25 mg/kg and 50 mg/kg did not significantly affect the locomotor activity. Beyond the aforementioned, gas chromatography-mass spectrometry analysis revealed the presence of the compound in the blood and brain after subchronic administration of citral. Taken together, the results obtained in vitro, in vivo and ex vivo clearly indicate that citral is a promising monoterpene that can potentially be used towards cognition improvement.

## 1. Introduction

**Figure 1:**
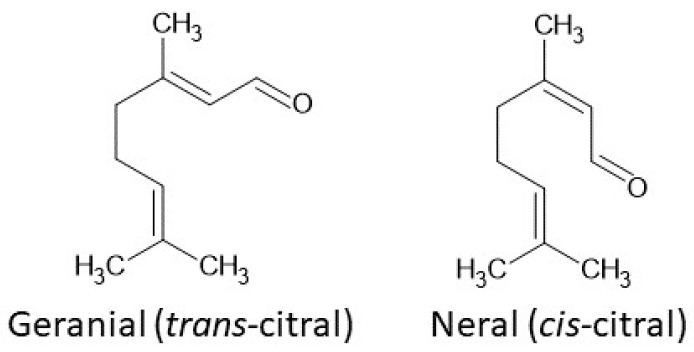
Structure of citral isomers: geranial (*trans*-citral) and neral (*cis*-citral).

Citral (3,7-dimethyl-2-6-octadienal) (Figure 1) is a volatile unsaturated aldehyde belonging to the largest group of secondary plant metabolites, namely terpenes. This monoterpene has been subjected to detailed study and is commonly used as a fragrance and flavoring, due to its characteristic odor. It can be found in numerous plants, e.g, A. citriodora, P. citriodora and Cymbopogon varieties [1,2]. The open-chain monoterpenoid, both natural and synthetic, is a mixture of isomers: cis-neral and trans-geranial. It is known that geranial is structurally similar to all-trans retinaldehyde. There are study results which indicate that citral is an antagonist of vitamin A, acting by inhibiting the oxidation of retinal to retinoic acid [3].

This compound has been revealed to be involved in numerous pro-health activities (antioxidant, antimicrobial, acetylcholinesterase (AChE) inhibitory, anti-inflammatory, chemo-preventive, learning improvement properties, etc.) [3,4,5]. Despite the significant pro-health side effects of the monoterpenoid, there are also results presenting pathological processes which are associated with citral activity, such as cutaneous anaphylaxis and prostatic hyperplasia [6]. Nevertheless, it is worth mentioning that citral is Generally Regarded as Safe (GRAS) by the US Food and Drug Administration, hence, this compound is considered safe for human and animal consumption [7]. Detailed in vivo studies based on rat embryos revealed that citral is embryotoxic only after topical, intraperitoneal or oral treatment at dose levels that were also toxic for mothers [3].

The presented paper is focused on the determination of citral’s influence on neurodegeneration symptoms (which, in most cases, are associated with behavioral disturbances). It is known that dementia, as well as disorders strictly associated with it (i.e., Alzheimer’s disease), may manifest themselves as minor disturbances related to memory and orientation in space, which deepen along with disease progress. Most of the neurological changes are multifactorial and therefore it is extremely important to find a substance that will be active in several possible directions [8]. In the case of Alzheimer’s disease (AD), the most important factors are oxidative stress, low level of neurotransmitters (i.e., acetylcholine), neurofibrillary tangles and amyloid β accumulation, disturbances to the levels of ion metals including Fe(II), Fe(III), Cu(II), Ca(II) and neuroinflammation [9].

Increasing attention is being paid to disease prevention based on following a healthy lifestyle and practicing proper nutrition by consuming a diet rich in plant products and natural compounds. To date, several drugs have been approved for the treatment of neurodegenerative diseases. Among them are drugs accepted for AD treatment, namely galantamine, donepezil, rivastigmine, memantine and aducanumab [10]. Nevertheless, their use is also associated with serious side effects, including hepatotoxicity [11]. Thus, alternatives are desired.

An analysis of available study results revealed that citral can be a good multi-directional agent against neurodegeneration and/or prevent the changes. Citral has been studied both in vitro and in vivo towards various neurodegeneration factors, but in vivo study results are limited. Among the in vitro assays, there are results pointing towards free radical scavenging activity, Fe(III) and Cu(II) reduction and Fe(II) chelation. Studies have revealed satisfactory activity towards all of the aforementioned factors [12,13,14]. Additionally, in vitro and in silico analyses of the monoterpene towards AChE have indicated the ability of citral to counteract low levels of neurotransmiter acetylcholine [15]. Despite research into the interesting and significant activity of citral in in vitro conditions, studies based on animal models are limited [16,17,18].

The presented paper is intended to enrich our current knowledge of the anti-neurodegenerative activity of citral as evidenced through mice models, as well as its butyrylcholinesterase (BChE) inhibitory activity, hepatotoxicity, detection of oxidative stress and lipid peroxidation using hepatic cells.

## 2. Results

### 2.1. BChE Inhibitory Activity

**Figure 2:**
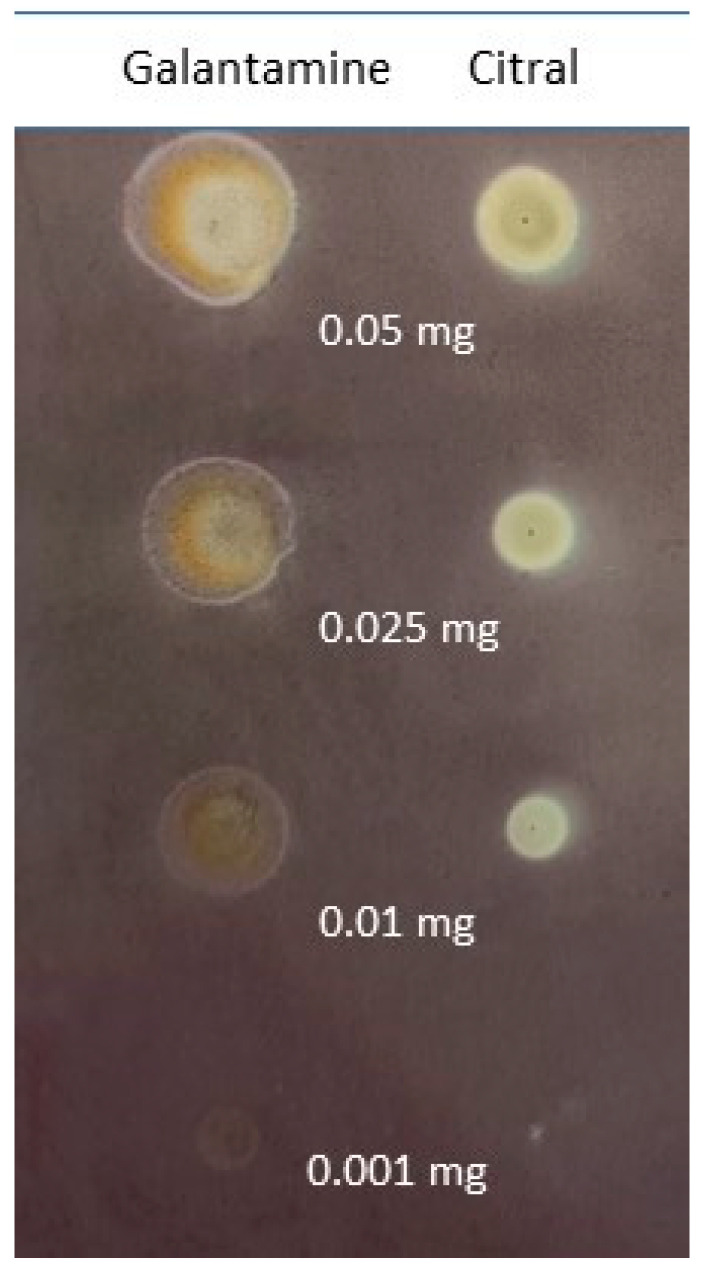
Inhibitory activity of citral and standard (galantamine) at various amounts: 0.05 mg, 0.025 mg, 0.01 mg and 0.001 mg.

Thin-layer chromatography was employed to evaluate citral’s BChE inhibitory activity. The use of a slightly modified Marston method [19] allowed us to determine the activity of various concentrations of citral and galantamine-a known AChE and BChE inhibitor. Figure 2 presents the obtained results.

The results were analyzed using the Sorbfil TLC Videodensitometer program which allows the activity of compounds to be assessed based on changes in the color of the spots with the applied compound compared to the background of the plate that does not contain active compounds. Additionally, comparative analysis allows the obtained results to be compared to the activity of the reference substance, which, in this case, is galantamine. Table 1 shows the BChE inhibitory activity (%) of citral in comparison to galantamine (activity assumed as 100%) for selected amounts of terpene.

In accordance with Table 1, citral was revealed to show satisfactory activity towards BChE inhibition. Interestingly, its activity did not change linearly with changing concentration, although it was expected that the activity would decrease as the amount of citral applied was reduced. Instead, the performed assay revealed a non-linear concentration–activity relationship for which the highest activity turned out to be for 0.01 mg of applied compound, whereas the highest and the lowest amounts of citral revealed the weakest activity.

### 2.2. In Vitro Hepatotoxicity of Citral

**Figure 3:**
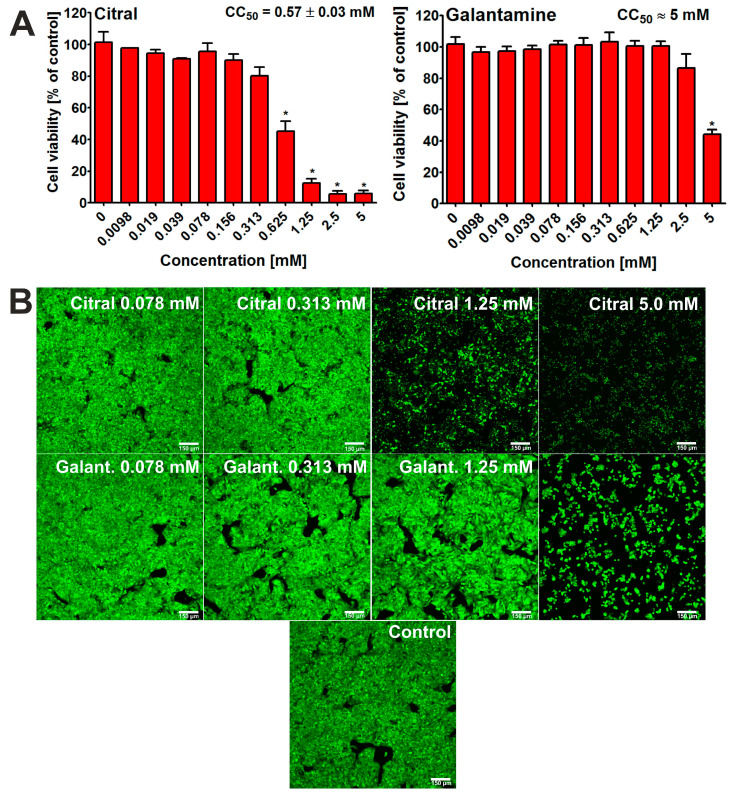
Viability of human hepatocellular carcinoma cells (HepG2 cell line, HB-8065) after 48 h incubation with citral and galantamine. MTT assay was used for quantitative evaluation of cytotoxicity (**A**); * statistically significant differences between cell viability after treatment with citral/galantamine and cell viability after incubation without tested substance (control, 0 mM), Student *t*-test, *p* < 0.05. Moreover, Live/Dead Double staining was used for visualization of live (green fluorescence) and dead cells (red fluorescence) (**B**); magnification 100×, scale bar = 150 μm.

In this study, the HepG2 cell line was used, as it is considered to be a model cell line for hepatotoxicity evaluation in searching for new potential drugs for treating neurodegeneration [20,21]. After 48 h of incubation, the 3-[4,5-dimethylthiazol-2-yl]-2,5 diphenyl tetrazolium bromide (MTT) assay [22] showed that citral possessed hepatotoxic activity at some tested concentrations (Figure 3A). Thus, the CC_50_ value for citral was determined at approx. 0.57 mM. This result was confirmed by CLSM observations (Figure 3B). In this case, cells treated only with selected concentrations were observed and photographed. Nevertheless, it is clearly evident that the viability of hepatocytes incubated with citral at concentration of 1.25 mM and 5 mM is significantly decreased. In turn, galantamine (reference compound, positive control) exhibited a lower hepatotoxocity compared to citral, with a CC_50_ value close to 5.0 mM (Figure 3A,B).

### 2.3. In Vitro Hepatoprotective Activity of Citral

**Figure 4:**
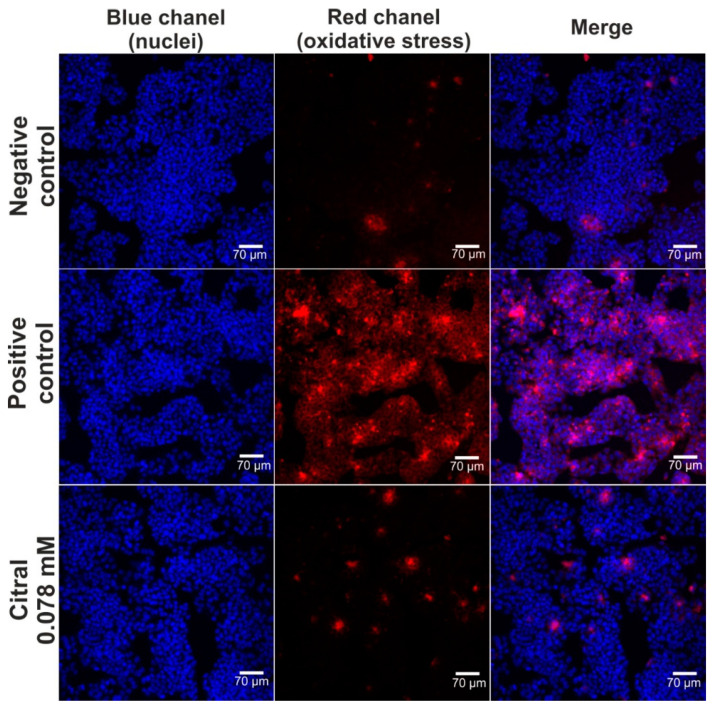
The CLSM images presenting hepatoprotective activity of citiral against H_2_O_2_-induced oxidative stress. Human hepatocellular carcinoma cells (HepG2 cell line, HB-8065) were incubated with citral for 48 h, followed by 1-h incubation with 500 μM H_2_O_2_. In turn, cells incubated only with culture medium are served as negative control, while cells incubated with culture medium, followed by incubation with 500 μM H_2_O_2_ are served as positive control. Cells were stained with Hoechst 33342 for nuclei visualization (blue fluorescence) and with CellROX^TM^ Deep Red Reagent for visualization of oxidative stress (red fluorescence); magnification 200×, scale bar = 70 μm.

**Figure 5:**
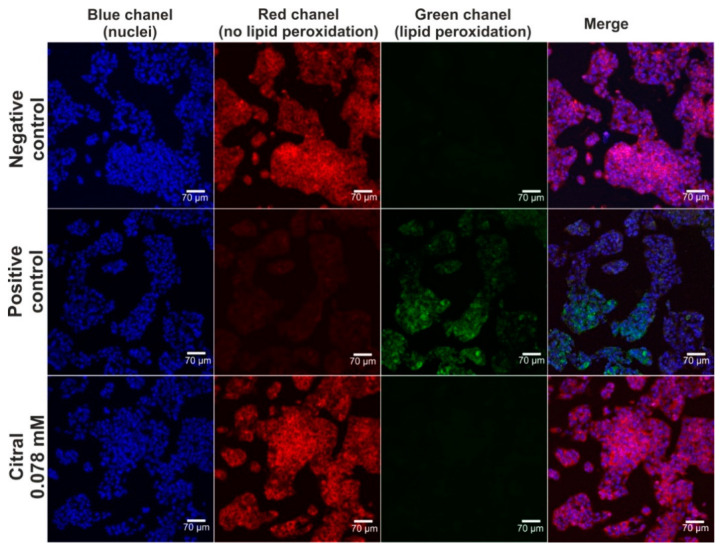
The CLSM images presenting hepatoprotective activity of citiral against H2O2-induced lipid peroxidation. Human hepatocellular carcinoma cells (HepG2 cell line, HB-8065) were incubated with citral for 48 h, followed by 1-h incubation with 500 μM H_2_O_2_. In turn, cells incubated only with culture medium served as negative control, while cells incubated with culture medium, followed by incubation with 500 μM H_2_O_2_ served as positive control. Cells were stained with Hoechst 33342 for nuclei visualization (blue fluorescence) and with Image-IT^TM^ Lipid Peroxidation for visualization of lipid peroxidation (green fluorescence) or lack of lipid peroxidation (red fluorescence); magnification 200×, scale bar = 70 μm.

The hepatoprotective activity of citral against H_2_O_2_-induced oxidative stress and lipid peroxidation was assessed via CLSM observations (Figure 4 and Figure 5). Based on data obtained from the MTT assay (previous experiment—in vitro hepatotoxicity of citral), a citral at concentration of 0.078 mM was used. Firstly, CLSM observations showed that citral significantly decreased the generation of ROS by H_2_O_2_ in HepG2 cells (Figure 4). As can be seen, H_2_O_2_ possessed a strong oxidative potential in HepG2 cells pre-incubated only with culture medium (positive control). These observations were also confirmed during staining (Figure 5), where ROS generated by H_2_O_2_ induced strong lipid peroxidation in HepG2 cells pre-incubated only with culture medium (positive control). In turn, citral protected HepG2 cells against lipid peroxidation. Hence, these observations indicated that this monoterpene has hepatoprotective potential in vitro.

### 2.4. The Impact of Acute and Subchronic Administration of Citral on Memory Acquisition Processes in the Passive Avoidance (PA) Test

**Figure 6:**
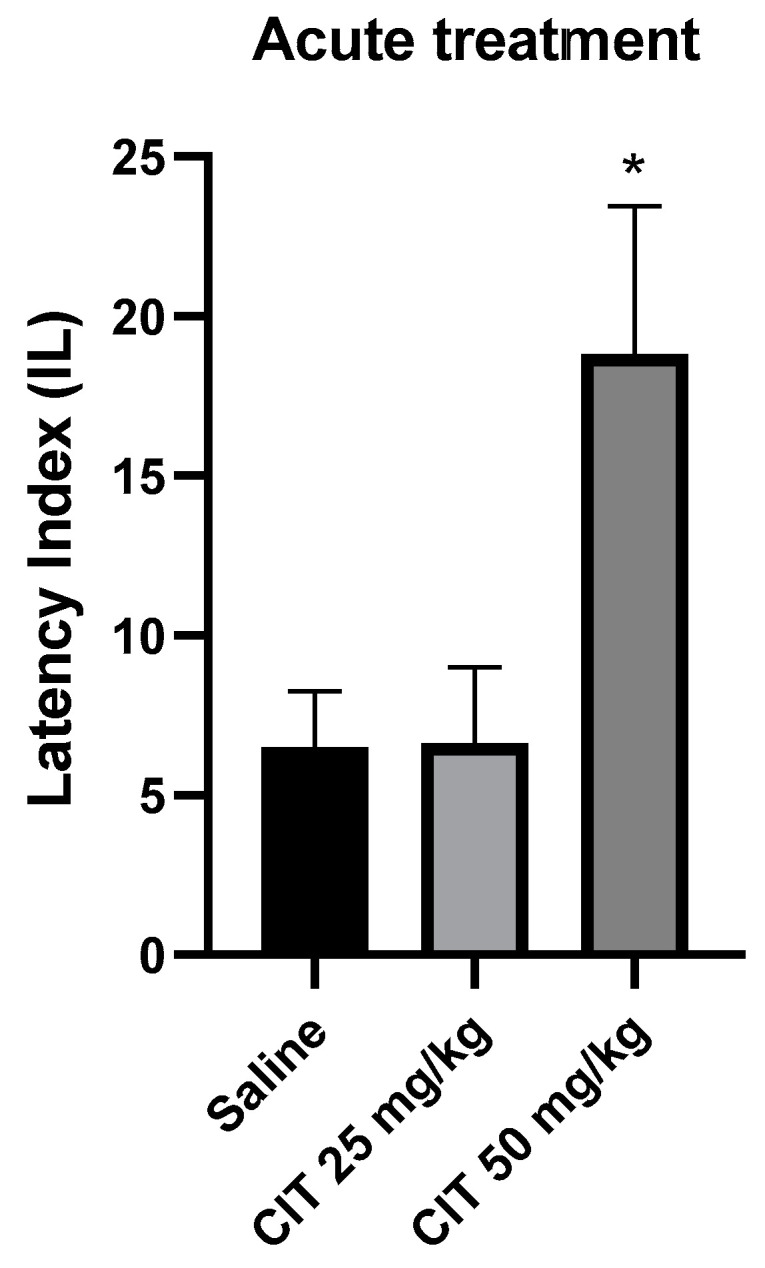
The effect of acute administration of citral (CIT) on the memory acquisition trials using the PA test in mice. CIT (25 or 50 mg/kg i.p.) or saline solution (i.p.) were administered 30 min before training. Subsequently, 24 h after training, the animals were re-tested. Results are expressed as latency index (IL) and presented as mean ± SEM, *n* = 8–10. Statistical analysis of data was performed using one-way analysis of variance (ANOVA), followed by the post hoc Dunnett’s test. * *p* < 0.05 vs. saline-treated control group.

One-way ANOVA revealed that acute administration of citral influenced the IL in the context of memory acquisition processes in the PA test [F (2, 24) = 5.085; *p* = 0.0144]. The Dunnett’s post hoc test showed a significant increase in IL after the injection of citral at a dose of 50 mg/kg compared to the control group receiving saline solution, indicating an improvement in memory processes associated with acquisition [*p* < 0.05]. Citral at a dose of 25 mg/kg did not change the IL compared to the saline-treated control group (Figure 6).

**Figure 7:**
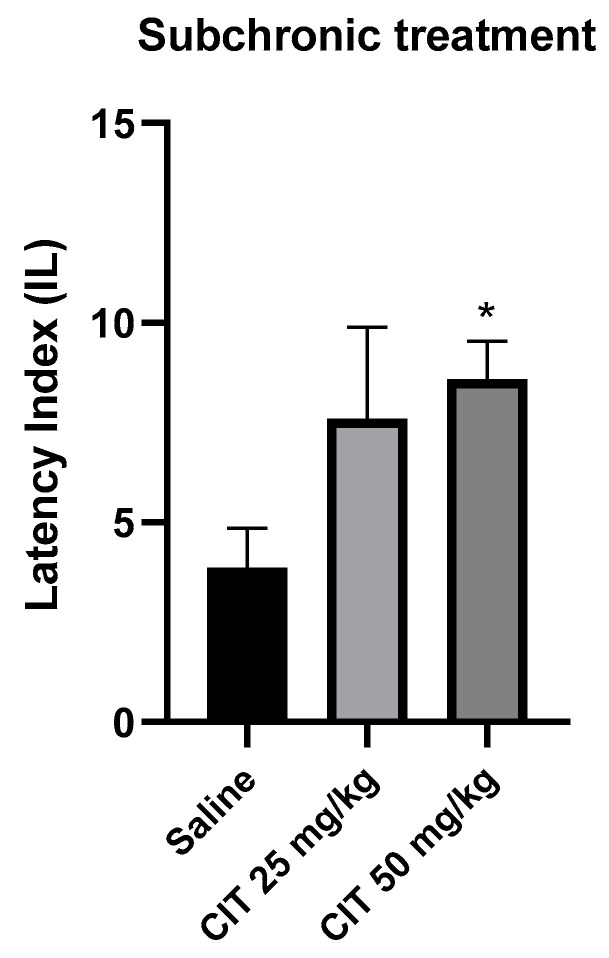
The effect of subchronic administration of citral (CIT) on the memory acquisition trials using the PA test in mice. CIT (25 or 50 mg/kg, i.p.) or saline solution (i.p.) were administered for 6 days, twice daily. On the seventh day, the appropriate group of mice received CIT (25 or 50 mg/kg, i.p.) or saline solution (i.p.) 30 min before training. Twenty-four hours after training, the animals were tested again. Results are expressed as latency index (IL) and presented as mean ± SEM, *n* = 9–10. Statistical analysis of data was performed via nonparametric Kruskal–Wallis ANOVA test, followed by the post hoc Dunn’s test. * *p* < 0.05 vs. saline-treated control group.

The nonparametric Kruskal–Wallis ANOVA test revealed that subchronic administration of citral influenced the IL in the context of memory acquisition processes in the PA [F (2, 26) = 2.387; *p* = 5.040]. The Dunn’s post hoc test showed a significantly increased IL after the repeated injection of citral at a dose of 50 mg/kg, as compared to the control group receiving saline solution, indicating an improvement in memory processes associated with acquisition [*p* < 0.05]. Citral at a dose of 25 mg/kg increased the IL value, but it did not reach the level of statistical significance (Figure 7).

### 2.5. The Impact of Acute and Subchronic Administration of Citral on Memory Acquisition Processes Impairment by an Acute Injection of Scopolamine in the Passive Avoidance (PA) Test

**Figure 8:**
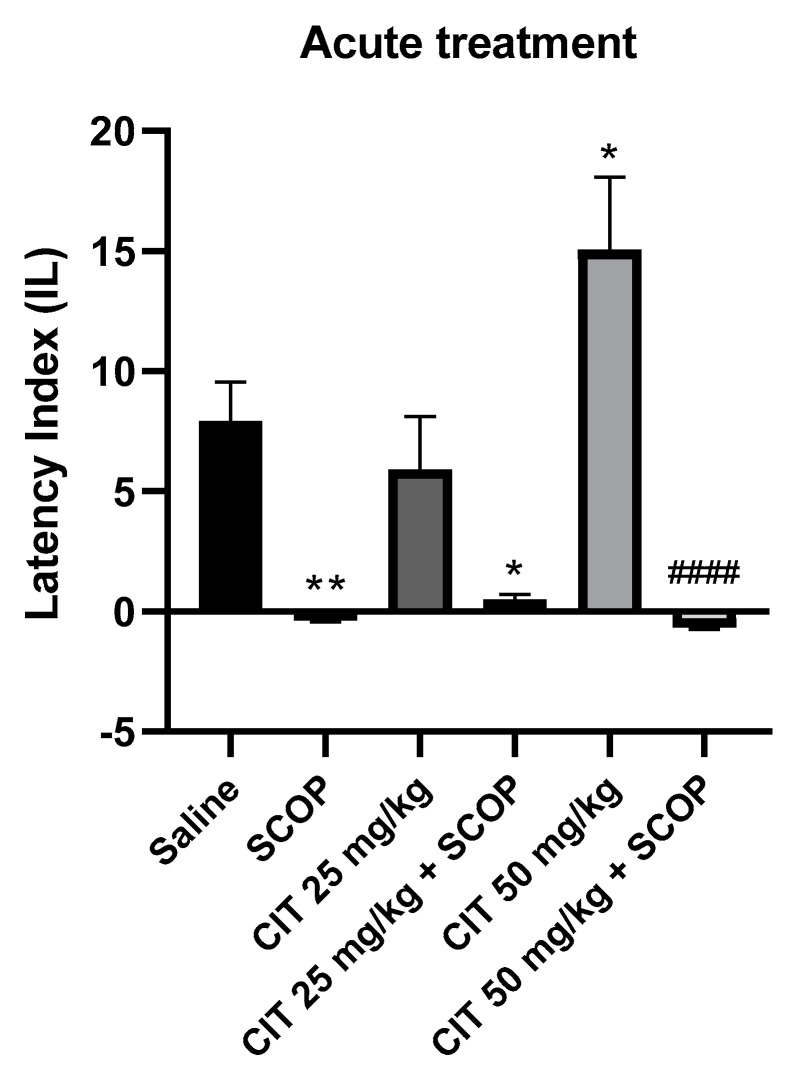
The effect of acute administration of citral (CIT) in scopolamine−induced impairment of memory acquisition trial using the PA test in mice. CIT (25 or 50 mg/kg, i.p.) or saline solution (i.p.) or scopolamine (1 mg/kg, s.c.) were administered 30 min before training. Subsequently, 24 h after training, the animals were tested again. Results are expressed as latency index (IL) and presented as mean ± SEM, *n* = 7–10. Statistical analysis of data was performed by employing two−way analysis of variance (ANOVA), followed by the post hoc Bonferroni’s test. * *p* < 0.05; ** *p* < 0.01 vs. saline-treated control group; ^####^ *p* < 0.0001 vs. CIT 50.

Two-way analysis of variance (ANOVA) showed statistically significant changes after scopolamine pretreatment [F (1, 46) = 50.87; *p* < 0.0001] and citral treatment [F (2, 46) = 3.253; *p* = 0.0477], while interactions between scopolamine and citral were observed [F (2, 46) = 4.850; *p* = 0.0123] (Figure 8). The post hoc Tukey’s test confirmed that scopolamine (1 mg/kg) treatment impaired the acquisition of memory [*p* < 0.01]. An improvement in memory acquisition induced by citral at a dose of 50 mg/kg was observed [*p* < 0.05]. There was a statistically significant difference in the IL values between the group treated with citral 50 mg/kg and the group treated with citral 50 mg/kg with scopolamine [*p* < 0.0001]. However, no significant statistical differences in the IL values were observed between the groups receiving citral (25 or 50 mg/kg) with scopolamine compared to the group receiving scopolamine alone, indicating that citral at doses of 25 and 50 mg/kg did not prevent the amnestic action of scopolamine.

**Figure 9:**
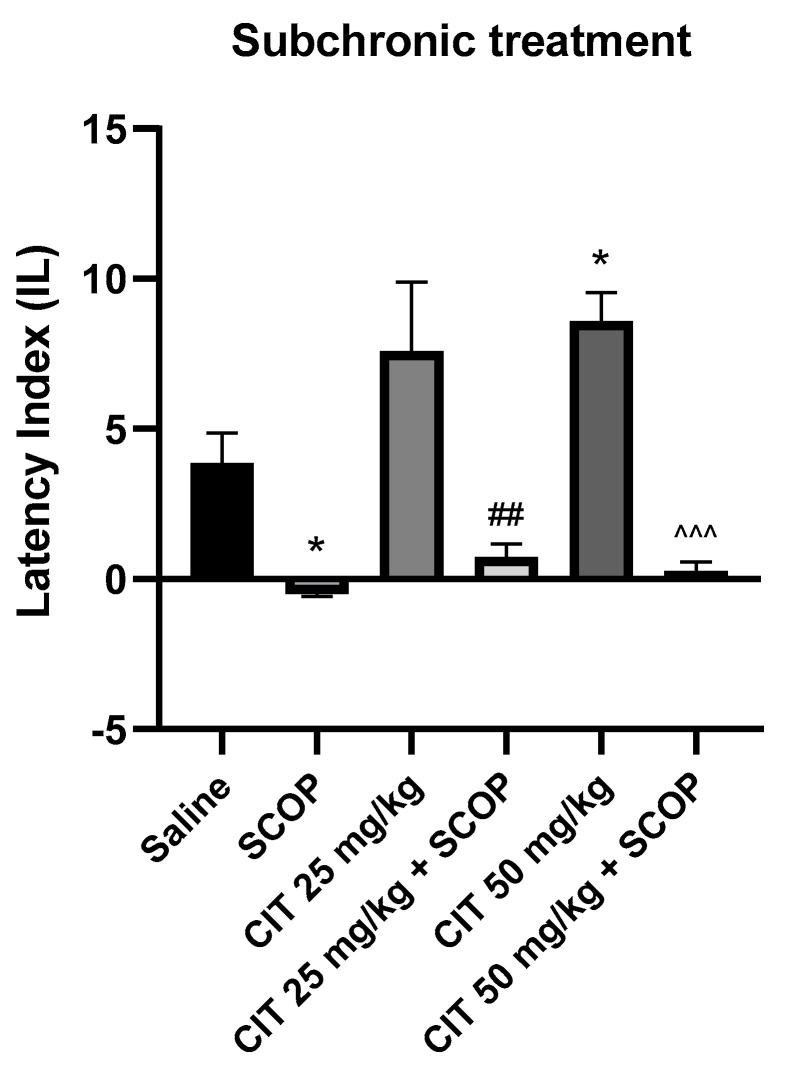
The effect of subchronic administration of citral (CIT) in scopolamine−induced impairment of memory acquisition trial using the PA test in mice. CIT (25 or 50 mg/kg, i.p.) or saline solution (i.p.) were administered for 6 days, twice daily. On the seventh day, the appropriate group of mice received CIT (25 or 50 mg/kg, i.p.) or saline solution (i.p.) or scopolamine (1 mg/kg, s.c.) 30 min before training. The animals were retested 24 h after training. Results are expressed as latency index (IL) and presented as mean ± SEM, *n* = 9–10. Statistical analysis of data was performed using two−way analysis of variance (ANOVA), followed by the post hoc Bonferroni’s test. * *p* < 0.05 vs. saline−treated control group; ^##^ *p* < 0.01 vs. CIT 25; ^^^^^ *p* < 0.001 vs. CIT 50.

Two-way ANOVA showed statistically significant changes after scopolamine pretreatment [F (1, 50) = 45.59; *p* < 0.0001] and citral treatment [F (2, 50) = 3.226; *p* = 0.0481], while no interactions were observed between scopolamine and citral [F (2, 50) = 1.416; *p* = 0.2523] (Figure 9). The post hoc Bonferroni’s test confirmed that scopolamine (1 mg/kg) treatment impaired the acquisition of memory [*p* < 0.05]. An improvement in memory acquisition induced by citral at a dose of 50 mg/kg was observed [*p* < 0.05]. Significantly different results were obtained for the groups receiving citral (25 or 50 mg/kg) compared to animals receiving citral (25 or 50 mg/kg) with scopolamine [*p* < 0.01; *p* < 0.001, respectively]. However, no statistically significant differences were found between the groups receiving citral (25 or 50 mg/kg) with scopolamine and the groups receiving only scopolamine, indicating that citral at these doses does not reverse the adverse effects of scopolamine on the acquisition of memory.

### 2.6. The Effect of Citral on Locomotor Activity in Mice

One-way ANOVA analysis showed that neither single [F (2, 21) = 0.9401; *p* = 0.4064] nor repeated [F (2, 21) = 0.6018; *p* = 0.5570] administration of citral at doses of 25 and 50 mg/kg affected the locomotor activity of mice measured for 30 min after injections. Similarly, during 60 min, acute [F (2, 20) = 0.8448; *p* = 0.4444] and repeated [F (2, 21) = 1.543; *p* = 0.2371] administration of citral in these doses did not significantly affect the tested parameter (Table 2).

### 2.7. GC-MS Analysis of Citral in Biological Material–Plasma, Hippocampus and the Rest of the Brain

GC-MS analysis showed the presence of cis-citral in all analyzed samples of biological material collected from mice after subchronic administration of the monoterpene at a dose of 50 mg/kg, which turned out to be most efficient in behavioral studies (Table 3). It should be noted that trans-citral was not detected in the analyzed tissues.

The obtained results confirm that citral is able to enter the brain and simultaneously cross the blood–brain barrier. Citral was detected in all analyzed tissues after subchronic administration. It should be emphasized that the amount of the monoterpene in hippocampus was much greater than in the rest of the brain. Analysis with use of *t*-test showed that the difference is statistically significant (*p* < 0.0001). Unfortunately, the monoterpene was not identified in biological samples after acute administration. However, if citral was detected after semi-chronic administration, it is highly probable that chronic administration will increase the amount of citral in the examined tissues.

## 3. Discussion

The neurological activity of citral was evaluated using various methods, both in vitro, in silico, in vivo and ex vivo. Due to the compound showing a wide range of biological activity, it is highly probable that it can act as an anti-neurodegenerative agent [23]. Despite a wide range of activity, in current literature, detailed in vivo analysis is limited, thus any approach to research with an animal model is important to expand our knowledge about this interesting natural compound.

Considering the results obtained for assays associated with neurodegeneration, citral can be considered as a potential candidate for a more detailed analysis of its effect upon dementia and neurodegeneration. In our work, a detailed in vitro Marston assay [19] revealed the strong ability of the monoterpene to inhibit BChE—one of the enzymes strictly associated with neurodegeneration. Interestingly, a non-linear activity–concentration relationship was observed. The highest activity, 131% in comparison to reference galantamine (100% activity), was observed for 0.01 mg of compound being applied onto the plate, whereas 0.05 mg induced a 44% inhibition of the enzyme.

Potential drugs should primarily be effective in non-cytotoxic doses. In our previous study, we preliminary evaluated the significance of citral as a potential drug for the treatment of Alzheimer’s disease [15]. Among many analyses, we also assessed cytotoxicity towards two model normal cell lines. Our research indicated that CC_50_ values for citral were 0.076 ± 0.012 mM (normal skin fibroblasts) and 0.136 ± 0.012 mM (normal kidney cells) [15]. Nevertheless, it is also important to evaluate the influence of drugs on hepatocytes, as they play crucial role in drug metabolism. It is known that drugs may become less toxic or even more toxic for cells after metabolic transformation [20]. The research conducted by other researchers indicated that even low concentrations of citral can induce cytotoxicity and genotoxicity in hepatocytes [24]. Our experiment, however, showed that this monoterpene did not exhibit hepatotoxicity (cell viability ≥ 95%) at low concentrations (0.0098–0.078) mM. It is also worth noting that although citral showed higher in vitro hepatotoxicity than galantamine, this monoterpene, unlike galantamine, has a broader spectrum of biological activities [12,13,14].

Effective drugs, in many cases, may cause hepatotoxicity. However, the mechanisms of drug-induced hepatotoxicity are poorly understood. Published research demonstrates that drug metabolism may lead to the creation of reactive metabolites that may cause oxidative damage to cells induced by generated ROS [25]. According to available literature data, citral not only does not cause oxidative damages in cells, but it also has protective properties against the effects of ROS [26,27]. This phenomenon is crucial and allows citral to be considered as a promising agent for counteracting and treating neurodegeneration [28].

In order to evaluate the influence of citral on locomotor activity and memory, in vivo tests based on a mice model were conducted. It should be emphasized that some studies were conducted to assess the effect of citral on cholinergic transmission using scopolamine. Scopolamine is a known blocker of muscarinic cholinergic receptors (mAChRs) (subtypes; M1 and M2) which manifests its effects in short-term and long-term memory disturbances [29]. In accordance with Giridharm et al. [30], scopolamine administration leads to significant reduction of acetylcholine level in mice. It should be recalled that scopolamine-induced amnesia is linked with oxidative stress and disturbance in the metabolism of low molecular weight antioxidants including glutathione. Such changes can have a significant impact on the high levels of lipid peroxidation that occur within the brain [31]. The mice model based on scopolamine-induced amnesia is preferred in studies of memory dysfunction, especially in testing new substances for their anti-AD properties.

Detailed analysis of passive avoidance test results revealed that citral is able to improve memory both in acute and subchronic administration. In both cases, the most effective dose was found to be 50 mg/kg—which improved memory with statistical significance. The lower dose (25 mg/kg) was also revealed to have a positive impact on IL value in subchronic administration; nevertheless, it did not reach the level of statistical significance. It should be emphasized that citral did not change the effects of scopolamine, and scopolamine eliminated the beneficial effect of citral on memory processes. In the context of the study and knowledge of the scopolamine mechanism, the active substance in this test should have an impact by increasing the ACh levels in the brain, along with strong antioxidant activity, which together would reverse the effect of scopolamine.

It is difficult to clearly answer the question of whether citral can act in the mechanism of AChE and BuChE inhibition. It should be taken into account that the scopolamine used in the studies is one of the most potent muscarinic receptor antagonists. The affinity of scopolamine for muscarinic receptors is <1 nM and is similar to ACh. Our study demonstrated the activity of citral in inhibiting AChE [15] and BuChE in vitro. We then found a procognitive effect of the tested compound, which was abolished by the scopolamine injection. These data suggest that the effect of citral on memory may be related to cholinergic transmission, but its effects at the doses used are not strong enough to reverse the adverse effects of scopolamine on memory acquisition. The results obtained in the passive avoidance test for citral and for citral in combination with scopolamine may also suggest a mechanism of action in the field of memory other than cholinergic.

A significant aspect of the activity in in vivo conditions is the influence of the examined compound on locomotor activity. Our work demonstrated that the administration of citral in 25 mg/kg and 50 mg/kg doses did not significantly affect the tested parameter.

GC-MS analysis of citral (sum of isomers) allowed us to detect only cis-citral. Citral was detected in all tissues collected from mice after subchronic administration of the monoterpene at dose of 50 mg/kg. The results indicate that citral crosses the blood–brain barrier and accumulates in the brain, especially in the hippocampus, which is involved in memory, learning and emotions [32]. Taking into account that the ‘trans’ isomer was not detected in the GC-MS analysis, it is important to assume that the ‘cis’ isomer is responsible for the positive effect on memory observed in the passive avoidance test.

In vitro activity towards various enzymes linked with dementia was evaluated. Among these, there is acetylcholinesterase (AChE), for which citral revealed 26.02% of inhibition at concentration 3.5 mM (Ellman assay), and satisfactory activity in the Marston assay (activity obtained for 0.01 mg of compound applied onto the plate) [15]. Additionally, citral activity was evaluated using molecular docking simulation (AutoDock and Molegro Virtual Docker), which revealed the compound’s interactions (H-bonds) with amino acid residues in close contact (up to 4 Å) with the AChE active site [15]. What is more, citral and essential oils rich in this monoterpene were analyzed in detail for their role in AChE inhibition. An example of which is the extract from leaves of C. limon that revealed in vitro and in vivo (Swiss mice) activity towards enzyme inhibition (approx. 30%), in comparison to neostigmine [28].

Besides AChE inhibitory ability, citral also reveals various other activities against neurodegeneration, i.e., antioxidant, Fe(III) reduction ability, impacts on the concentration of brain-derived neurotrophic factor (BDNF) and malondialdehyde (MDA), etc. [33]. Previous research points to the various possible mechanisms of action of citral, which are based on the in vivo results obtained. A representative case is that presented by Beniwal et al. [33], who presented an improvement in behavioral responses and a decrease in anxiety and depression in rats as a result of citral administration. In this work, the activity of the monoterpene was found to result from an increase in BDNF and an improvement in the antioxidant–oxidant status. The inhibitory activity of citral in retinoic acid synthesis is equally interesting [3]. It is known that retinoic acid is responsible for a strong enhancement effect on acetylcholine (ACh) transmitter functions in brain cholinergic neurons [34]. It has been shown that low concentrations of citral almost completely inhibits retinoic acid formation. The sensitivity is demonstrated by all three isozymes of human aldehyde dehydrogenase [35].

In analyzing the activity of citral, various concentrations were studied. Of significance is that the biphasic effect of the monoterpene was found to depend on its concentration when observed in animal models. The effect was first observed by Yang et al., who analyzed the impact of citral on spatial learning and memory in rats [6]. In our work, the results revealed opposing activity of the monoterpene depending on dosage. In the case of 0.1 mg/kg, citral had an explicit influence on the spatial learning capabilities and enhanced the spatial reference memory of rats. The opposite effect was observed for a dose of 1 mg/kg. The effect has been associated with the retinoic acid concentration in the hippocampus, namely, a low dose of citral brought about an increase in the retinoic acid concentration, whereas a high dose decreased it.

One of the common symptoms of cognitive impairment is anxiety, which can be observed in most patients with AD and 10% to 45% of all patients with mild cognitive impairment (MCI) [36]. The activity of citral towards this symptom was also analyzed in our study. In the paper presented by Moghaddam et al. [7], intraperitoneal administration of citral resulted in a positive effect on anxiety (elevated plus maze test, open field test), whereas the highest activity was observed for a dose of 20 mg/kg, but, simultaneously, lower dosages of 5 and 10 mg/kg also caused significant increase in anxiolytic effect in comparison to control. It is probable that citral, besides having impact on retinoic acid, can act via GABAA and 5-HT1A receptor modulation [7].

It is worth mentioning that citral can be quickly eliminated and does not bioaccumulate in the body; hence, a direct evaluation of its impact on spatial memory by determining the level of the monoterpene in organs is very difficult to undertake [6]. Citral is an unstable and hydrophobic compound under normal storage conditions, so it can easily be degraded and lose its pharmaceutical properties [37]. This known effect can explain the trace amounts of citral in the tested ex vivo samples.

In analyzing the in vivo activity of citral, it is important to underline that an extremely small number of studies have been aimed at determining the concentration of citral or its metabolites in various organs in animal models. In most cases, the activity of the monoterpene in animal models is estimated based on behavioral tests, enzyme activity or level of selected parameters, and only a few papers have reported on the evaluation of level of citral (neral/geranial) or its potential metabolites in serum or selected organs. One was presented by Phillips et al. in 1976, who considered the issue of absorption, distribution and excretion of citral in rat and mouse models [18]. Herein, [14C] citral was administered to mice in the form of a compound dissolved in corn oil via oral intubation, and we provided a dose equal to 100 mg/kg. The results showed that the greatest proportion of radioactivity was absorbed after 12 h, but activity was not detected in the brain, thymus, eye lens and skeleton at this time, whereas activity in these parts was observed at 72 h. Moreover, nearly 90% of all radioactivity was eliminated within 72 h, and complete excretion was observed after 120 h.

## 4. Materials and Methods

### 4.1. In Vitro Assays

#### 4.1.1. Materials

The following reagents: citral (≥95%), butyrylcholinesterase, type V-S from Electrophorus electricus, albumin from bovine serum, 1-naphthyl acetate, Trizma^®^ (2-amino-2-(hydroxymethyl)-1,3-propanediol) hydrochloride solution (1 M, pH 7.8), acetylthiocholine iodide (≥99%), DTNB (5,5′-dithiobis(2-nitrobenzoic acid)), galantamine hydrobromide from Lycoris sp. (>94%) and Fast Blue B salt 95%, penicillin, streptomycin, Live/Dead Cell Double Staining Kit, Hoechst 33342 dye, layer chromatography plates (HPTLC silica gel 60), scopolamine hydrobromide, citral and Tween 20 were purchased from Sigma Aldrich (now, Merck) (St. Louis, MO, USA). HepG2 cells (HB-8065, human hepatocellular carcinoma cell line) and Eagle’s Minimum Essential Medium (EMEM) were supplied by ATCC (London, UK), while fetal bovine serum (FBS) was obtained from Pan-Biotech (Aidenbach, Germany). Additionally, CellROXTM Deep Red Reagent and Image-ITTM Lipid Peroxidation Kit were purchased from Invitrogen, ThermoFisher Scientific (Waltham, MA, USA). Solvents (analytical purity grade) were obtained from Polish Reagents (Gliwice, Poland).

#### 4.1.2. Methods

Butyrylcholinesterase (BChE) Inhibitory Assay on TLC Plates According to the Marston MethodThe assay was performed based on a slightly modified method first presented by Marston et al. [19]. The experiment was performed using chromatographic HPTLC silica gel 60 (F254) plates. The plates were first prepared by elution with ethanol and activation for 40 min at 105 °C. The next step was terpene application onto the plate by means of an automatic applicator, Desaga AS-30 (Biostep GmbH, Burkhardtsdorf, Germany). Due to the high volatility of the monoterpene, before application, citral was diluted 10 times in ethanol. The following amounts of citral were used [mg]: 0.05; 0.025; 0.01 and 0.001. The amount of the compound employed was decided on the basis of previously conducted tests for acetylcholinesterase [15]. Galantamine, a known AChE inhibitor, was chosen as a reference standard. Solution of the standard (1 mg/mL in ethanol) was applied onto the plates in the same amount as the investigated terpene. The next step of assay was performed in accordance with [15]. The activity was determined based on the discoloration of the dark background where the compounds were spotted. Obtained results were analyzed via the Sorbfil TLC Videodensitometer program (ver. 2.0).

##### Evaluation of Hepatotoxicity In Vitro

The experiments were performed using human hepatocellular carcinoma cells (HepG2 cell line). The cells were maintained according to manufacturer recommendations. Briefly, the cells were cultured in Eagle’s Minimum Essential Medium (EMEM) supplemented with 100 U/mL penicillin, 100 μg/mL streptomycin and 10% fetal bovine serum (FBS). The appropriate conditions for cell culture were 37 °C, 5% CO_2_ and 95% humidity. Before the experiment, the HepG2 cells were seeded onto 96-well plates at concentration of 3 × 10^4^ cells/well and incubated for 24 h at 37 °C (5% CO_2_, 95% humidity). Subsequently, serial dilutions (0.0098–5 mM) of citral or galantamine (reference compound, positive control) were prepared and added to the cells. After 48 h of incubation, the cell viability was evaluated using MTT assay, according to procedure described previously in detail [38]. For this reason, four independent experiments were conducted. The results were expressed as percentage of control (the OD for control cells incubated without tested compounds was considered as 100%). The results were presented as mean value ± standard deviation (SD). Student *t*-test was used to determine statistical differences between samples. Moreover, 4-parametric logarithmic curves were created in order to assess concentration that caused 50% decrease of cell viability (CC_50_ value) compared to control cells. In turn, to confirm quantitative results, the cells were stained with Live/Dead Cell Double Staining Kit, observed under confocal laser scanning microscope (CLSM), and photographed.

##### Detection of Oxidative Stress and Lipid Peroxidation

The experiment was carried out according to the same procedure as described in Section Evaluation of hepatotoxicity in vitro, but using citral at concentration of 0.078 mM. After 48 h of incubation, the solution was removed and oxidative stress was induced by 1 h treatment with 500 μM H_2_O_2_ prepared in culture medium. Cells incubated with a culture medium for 48 h, followed by 1 h without H_2_O_2_ served as negative control, while cells incubated with culture medium by 48 h followed by 1 h treatment with H_2_O_2_ served as a positive control. Subsequently, the cells were stained using CellROX^TM^ Deep Red Reagent or Image-IT^TM^ Lipid Peroxidation Kit, while cell nuclei were stained with Hoechst 33342 dye. CellROXTM Deep Red Reagent is a non-fluorescent dye in a reduced state, which after oxidation by reactive oxygen species (ROS), exhibits strong red fluorescence. In turn, Image-ITTM Lipid Peroxidation is a fluorescent dye enabling the highlighting of lipid peroxidation cells. After oxidation by ROS, this dye exhibits green fluorescence indicating lipid peroxidation, and gives red fluorescence when lipid peroxidation did not occur. The cells were observed under CLSM and photographed.

### 4.2. In Vivo Assays

#### 4.2.1. Preparation and Administration of Drugs

Scopolamine was dissolved in saline solution (0.9% NaCl) and administered subcutaneously (s.c.), while citral was suspended in a 1% solution of Tween 20 and administered intraperitoneally (i.p.). All substances were administered at a volume of 10 mL/kg, 30 min prior to memory tests or directly before locomotor activity assessment. Drug solutions were prepared daily immediately before use. The control group received injections of saline solution in the same volume and at the same time as the corresponding drugs. The doses of citral and scopolamine were selected based on published literature [16,20].

#### 4.2.2. Animals

The experiments were conducted on naive adult male Swiss mice, maintained in the Centre of Experimental Medicine of the Medical University of Lublin. The mice weighed between 20 and 25 g at the beginning of the study. Experiments were carried out between 8.30 a.m. and 4.00 p.m. The animals were housed in colony cages under standardized laboratory conditions: a natural 12/12 h light–dark cycle, temperature maintained at 21 ± 1 °C, air humidity between 50 ± 5%, with air exchange occurring at a rate of 15/h, and with free access to tap water and food (Altromin, Animalab, Lage, Germany). After a 7-day acclimatization period, the mice were randomly assigned to experimental groups consisting of 8–10 animals.

#### 4.2.3. Methods

##### Passive Avoidance Task

The passive avoidance (PA) task is based on the natural aversion of rodents to bright places and is considered a measure of long-term memory [39]. Depending on the testing protocol used, the PA test offers the opportunity to explore different memory stages based on the timing of drug administration. In our research, compounds were given prior to the initial trial (before the pretest), impeding the process of information acquisition.

The apparatus consisted of an acrylic box divided by sliding doors into two compartments: illuminated and darkened. The floor of the apparatus is made of stainless-steel bars serving as conductors for electric current.

**Figure 10:**
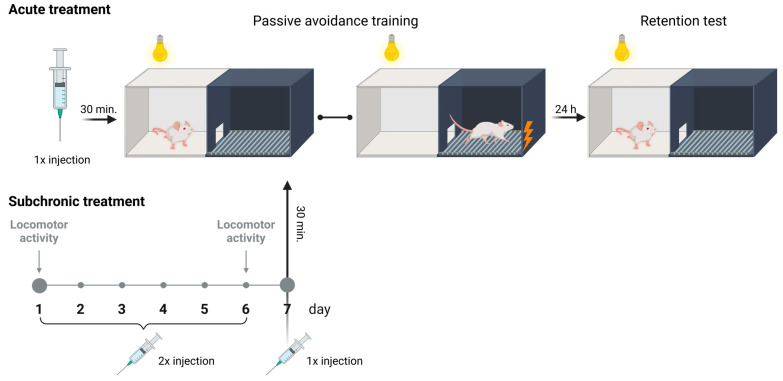
Graphical diagram of the experimental protocol used in the undertaken behavioral research created with BioRender.com.

On the first day of the experiment, the tested mice, after drug administration, were placed in the illuminated part of the apparatus and allowed to freely explore the illuminated chamber for 30 s with the doors closed. After this time, the sliding doors were raised, and they had access to the darkened chamber. Upon entry into the dark chamber with all four paws, the sliding doors were closed, and a weak electrical current (2 s exposure to shock, 0.2 mA) was delivered through the floor (Figure 10). The latency time for entry into the dark chamber was recorded as TL1. The observation period lasted for a maximum of 300 s. If the mouse did not enter the dark box during this time, it was placed in the dark box, the doors were closed, and a weak electrical current was delivered to the animal’s feet. In such cases, the TL1 value was recorded as 300 s.

On the following day of the test (24 h later), the same mice were again placed in the illuminated compartment of the apparatus with the sliding doors closed. After a 30 s adaptation period, the doors between the compartments were raised, and the time at which the animal entered the dark chamber was recorded as TL2. No electric shock stimulus was delivered in this trial (Figure 10). If the animal did not enter the dark chamber within 300 s, the test was terminated, and TL2 was recorded as 300 s [40].

##### Locomotor Activity

The locomotor activity scores were assessed using an Opto-Varimex-4 Auto-Track actimeter (Columbus Instruments, Columbus, OH, USA). The apparatus consists of a square plexiglass cage measuring (43 × 43 × 32 cm). Photoresistors and sensors are mounted on the wall at 2 heights (2.5 and 1.2 cm). When a moving animal crosses the beam, the change is recorded by the respective counter as one movement. The difference in counter readings determines the spontaneous mobility of the animals. Immediately after compound administration, the mice were individually placed in the actimeter for 60 min. The number of beam breaks by mice was recorded as locomotor activity at 30 and 60 min.

##### Treatment of Behavioral Research

The behavioral component of the experiment aimed to assess the influence of acute and subchronic administration of citral on memory acquisition in mice using the PA test. In the subsequent stage, an attempt was made to evaluate the mechanism of action of citral by examining its impact on memory acquisition processes following impairment by scopolamine [41].

To assess the impact of a single dose of citral on memory processes, animals were randomly assigned to 3 groups and administered the following substances: (1) saline solution (0.9% NaCl) i.p., (2) citral 25 mg/kg i.p. and (3) citral 50 mg/kg i.p. To evaluate the potential mechanism of action of citral, animals were randomly allocated to 6 groups and given the following compounds: (1) saline solution (0.9% NaCl) i.p., (2) scopolamine 1 mg/kg s.c., (3) citral 25 mg/kg i.p., (4) citral 25 mg/kg i.p. and scopolamine 1 mg/kg s.c., (5) citral 50 mg/kg i.p., (6) citral 50 mg/kg i.p. and scopolamine 1 mg/kg s.c. On the first day of the experiment, after administration of the test compounds, mice were trained in the PA test. All compounds were introduced 30 min before the pretest (assessment of memory acquisition processes). 24 h later, mice were retested in the PA test.

To assess the impact of subchronic administration of citral on memory processes, animals were randomly allocated to 3 groups: (1) control, (2) citral (25 mg/kg), (3) citral (50 mg/kg). For 6 days, twice daily (at 8:30 a.m. and 6:00 p.m.), and on the seventh day in the morning, animals received (1) saline solution (0.9% NaCl) i.p., (2) citral 25 mg/kg i.p. and (3) citral 50 mg/kg i.p., and 30 min after injection, animals were trained in the PA test. On the eighth day, mice were retested in the PA test, without injections.

To evaluate the potential mechanism of action of citral following subchronic administration, subsequent animals were randomly allocated into 6 groups: (1) control, (2) scopolamine (1 mg/kg), (3) citral (25 mg/kg), (4) citral (25 mg/kg) with scopolamine, (5) citral (50 mg/kg), (6) citral (50 mg/kg) with scopolamine. For the first 6 days of the experiment, animals from each group received the following compounds twice daily (at 8:30 a.m. and 6:00 p.m.): (1) and (2) saline solution (0.9% NaCl) i.p., (3) and (4) citral 25 mg/kg i.p., (5) and (6) citral 50 mg/kg i.p. On the seventh day in the morning, animals received the following injections: (1) saline solution (0.9% NaCl) i.p., (2) scopolamine 1 mg/kg s.c., (3) citral 25 mg/kg i.p., (4) citral 25 mg/kg i.p. and scopolamine 1 mg/kg s.c., (5) citral 50 mg/kg i.p., (6) citral 50 mg/kg i.p. and scopolamine 1 mg/kg s.c. (Table 4). On the seventh day of the experiment, 30 min after injection, animals were trained in the PA test. On the eighth day, the PA test was reapplied. Additionally, locomotor activity was measured in animals assigned to groups 1, 3 and 5. Animals were tested on the first and sixth days of the experiment following the morning injection.

##### Tissue Collection and Preparation

Immediately after the passive avoidance test (24 h after the last injection), the animals were decapitated. Blood and brains were collected. Blood was centrifuged at 13.000 rpm, 10 min., at +4 °C and the supernatant was collected. Brains were rinsed with an ice-cold saline solution, then hippocampi were isolated from the brains. The obtained biological material was stored at −80°C for further analysis. Tissues were homogenized in ultrapure water (1:2 *w/v* for remnants of the brains; 1:4 *w*/*v* for hippocampi) using a bead mill homogenizer (BeadBug 6, Benchmark Scientific, Sayreville, NJ USA) at 4000 rpm for 15 s. The homogenate was centrifuged at 10.000 rpm for 15 min at +4°C, and the supernatant was collected for further analysis.

#### 4.2.4. Ethical Declaration

All experiments were conducted following the ARRIVE guidelines and the European Community Council Directive for the Care and Use of Laboratory Animals of 22 September 2010 (2010/63/EU) and approved by the Local Ethics Committee in Lublin, Poland (Permission No: 42/2022).

### 4.3. Ex Vivo Assay

#### GC-MS Analysis of Citral in Biological Material–Plasma, Hippocampus and the Rest of the Brain

The GC–MS system consisted of a model 7890 A series gas chromatograph coupled with Detector Agilent Technologies 5975 C inert XL MSD with Triple–Axis Detector (Agilent, Wood Dale, IL, USA). Separation was performed on a HP–5MS column (30 m × 0.25 mm, 0.25 µm film thickness) supplied by Agilent Technologies (Wood Dale, IL, USA). The carrier gas was 99.99% high purity helium with a flow rate of 1.2 mL min-1. The sample volume was 1 µL. The injection port and the detector temperatures were 270 °C and 280 °C, respectively. Oven temperature program was initially set at 50 °C for 1 min, then ramped at 3 °C min-1 to 110 °C and held for 1.0 min, then ramped at 20 °C min^−1^ to 200 °C and held for 5 min. The total run time was 31.5 min with a solvent delay of 10 min. Ionization was performed in electron impact ionization (EI) mode at 70 eV. Selective ion monitoring (SIM) was set for quantification with dwell time 100 ms ion^−1^. Data were collected using Agilent MassHunter Quantitative Analysis Software–Perlan (ver. 05.02). Retention times of cis-citral, trans-citral and thymol (IS—internal standard) were 22.13 min, 23.10 min and 21.08 min, respectively. The characteristic ions (*m*/*z*) cis–citral and trans–citral were 152, 69, 41 and for thymol (*m*/*z*) z 150, 135, 91. The final concentration of the IS was 30 µg mL^−1^. Cis- and trans-citral contents were calculated from a commercial standard (Sigma-Aldrich, C83007-5ML) based on the area of the peaks obtained during chromatographic separation.

In this part of the experiment, 200 µL of hexane–ethyl acetate mixture (1:1) was added to the supernatant (from the collected biological material-plasma, hippocampus and remnant of the brain after separation of the hippocampus), with an internal standard so as to produce a final concentration of 30 ug/mL). The sample was shaken for 10 min to extract the active compounds by utilizing a vortex mixer. The sample was then centrifuged in a high-speed cryogenic centrifuge at 12,000 rpm for 10 min and the supernatant was transferred to a 1.5 mL clean autosample tube. The, 1 µL of the supernatant was injected into GC–MS system for analysis. Four samples of biological material were used for each analysis and tested three times. Concentrations of cis- and trans-citral in mice were calculated on the basis of calibration curves (Table 5).

### 4.4. Statistical Analysis

The data were presented as the mean ± SEM. Outliers were identified and excluded from the analysis by employing the ROUT method. The following tests were applied for statistical analysis of the obtained results: one-way analysis of variance ANOVA, followed by the post hoc Dunnett’s test; two-way analysis of variance ANOVA, followed by the post hoc Bonferroni’s test; nonparametric Kruskal–Wallis ANOVA test, followed by the post hoc Dunn’s test. The significance level was set at *p* < 0.05. The statistical analysis was conducted based on the nature of the data, with respect to best statistic practice in biomedical research [42].

To assess memory processes, a latency index (IL) was utilized, which was calculated for each animal. IL serves for the quantitative assessment of PA test performance as the disparity between retention and training latencies, and was computed as follows:

IL = (TL2 − TL1)/TL1

where: TL1 signifies the duration taken to enter the dark compartment during the training phase

TL2 indicates the period taken to re-enter the dark compartment during retention [20,23].

In order to determine statistical significance in the difference in citral content in the hippocampus and the rest of the brain, *t*-test was used.

## 5. Conclusions

Scientific studies have explicitly indicated the various biological activities of citral. Among these, some activities can be associated with neurodegeneration, and the positive effects of citral administration on this condition suggests that citral can be considered for use in preventing and treating neurodegeneration and dementia. The in vitro studies presented in this paper explicitly demonstrated the activity of citral towards BuChE inhibition, along with hepatoprotective properties. Moreover, citral did not reveal hepatotoxicity at low dose and simultaneously protected HepG2 cells against oxidative stress and lipid peroxidation. Hence, these observations indicate that this monoterpene has hepatoprotective potential in vitro. It is also worth mentioning that citral positively influenced memory after its acute and subchronic administration in vivo. The most effective rate of intake turned out to be a dose equal to 50 mg/kg, which simultaneously did not impact locomotor activity.

It is difficult to answer the question of whether citral can act in the mechanism of AChE and BuChE inhibition. It is known that the pro-cognitive activity of the monoterpene, with a high degree of probability, results from its cholinergic activity. However, based on the obtained results, this activity was not high enough to reverse the effect of the muscarinic receptor antagonist scopolamine. A mechanism of action of citral other than cholinergic is also possible. Additionally, the presence of cis-citral in the brain of the tested animals after subchronic administration of the monoterpene (mix of isomers) has confirmed the ability of citral to cross the blood-brain barrier. It should be emphasized that the presented analysis of citral in tissues collected from tested animals is one of the few available studies that showed the presence of this compound using direct tests.

## Figures and Tables

**Table 1:** BChE inhibitory activity [%] of citral and galantamine.

Compound	Inhibitory Activity [%] for Selected Amount of Compounds			
	0.05 mg	0.025 mg	0.01 mg	0.001 mg
Galantamine *	100	100	100	100
citral	44	63	131	35

* galantamine activity was assumed to be 100%.

**Table 2:** The effect of citral on locomotor activity in mice. In acute treatment, CIT (25 or 50 mg/kg, i.p.) or saline solution (i.p.) were administered once immediately before the spontaneous locomotor activity test. In repeated treatment, the same compounds were administered for 5 days, twice daily. On the sixth day, the appropriate group of mice received CIT (25 or 50 mg/kg, i.p.) or saline solution (i.p.) and were then immediately placed in actimeters. Locomotor activity (number of interruptions of light beams) was recorded for 30 and 60 min. Data are presented as means  ±  SEM, *n* = 7–8.

	Acute Treatment			Repeated Treatment		
	Control	CIT 25 mg/kg	CIT 50 mg/kg	Control	CIT 25 mg/kg	CIT 50 mg/kg
Photocell beam breaks ± SEM (30 min)	4950 ± 235.8	4260 ± 447.7	4865 ± 442.4	4686 ± 443.5	3784 ± 367.0	3799 ± 401.1
Photocell beam breaks ± SEM (60 min)	7938 ± 527.9	6860 ± 494.9	7946 ± 860.8	7721 ± 693.1	6143 ± 717.5	6312 ± 682.0

**Table 3:** Quantitative GC-MS analysis of cis-citral in tissues collected from mice after subchronic administration of the monoterpene at a dose of 50 mg/kg. Data are presented as the mean concentration ± SD [µg] in 1 mL of plasma or 1 mg of tissue from which citral was extracted. RSD%—relative standard deviation (*n* = 3). Statistical analysis of data was performed using *t*-test. *** *p*  <  0.001 vs. remnant of the brain.

	Tissues		
	Plazma [µg/mL]	Hippocampus [µg/mg of Tissue]	Remnant of the Brain [µg/mg of Tissue]
Average content	13.105	0.117 ***	0.02
SD	0.12	0.01	0.0003
RSD%	0.89	7.42	1.37

**Table 4:** Substance administration scheme to assess the potential mechanism of action of citral after subchronic administration. SCOP—scopolamine; CIT—citral.

Groups	Substances Administered from Day 1 to 6	Substances Administered on Day 7
(1) Control	saline solution (0.9% NaCl)	saline solution (0.9% NaCl)
(2) SCOP	saline solution (0.9% NaCl)	scopolamine 1 mg/kg s.c.
(3) CIT 25 mg/kg	Citral 25 mg/kg i.p.	Citral 25 mg/kg i.p.
(4) CIT 25 mg/kg + SCOP	Citral 25 mg/kg i.p.	Citral 25 mg/kg i.p. + scopolamine 1 mg/kg s.c.
(5) CIT 50 mg/kg	Citral 50 mg/kg i.p.	Citral 50 mg/kg i.p.
(6) CIT 50 mg/kg + SCOP	Citral 50 mg/kg i.p.	Citral 50 mg/kg i.p. + scopolamine 1 mg/kg s.c.

**Table 5:** Parameters of the calibration curve for cis- and trans-citral.

No.	Compound	Calibration Curve	R^2^
1	*cis*-citral	y = −0.0254426 x2 + 1.46612 x + 0.0137605	0.997
2	*trans*-citral	y = −0.0116753 x2 + 1.43904 x + 0.164956	0.997

## Data Availability

The research data are available from the authors.

## References

[1] Hirai M., Ota Y., Ito M. (2022). Diversity in Principal Constituents of Plants with a Lemony Scent and the Predominance of Citral. J Nat. Med..

[2] Rana V.S., Das M., Blazqeuz M.A. (2016). Essential Oil Yield, Chemical Composition, and Total Citral Content of Nine Cultivars of Cymbopogon Species from Western India. J. Herbs Spices Med. Plants.

[3] Di Renzo F., Broccia M.L., Giavini E., Menegola E. (2007). Citral, an Inhibitor of Retinoic Acid Synthesis, Attenuates the Frequency and Severity of Branchial Arch Abnormalities Induced by Triazole-Derivative Fluconazole in Rat Embryos Cultured in Vitro. Reprod. Toxicol..

[4] Ganjewala D., Gupta A.K., Muhury R. (2012). An Update on Bioactive Potential of a Monoterpene Aldehyde Citral. J. Biol. Act. Prod. Nat..

[5] Shi C., Song K., Zhang X., Sun Y., Sui Y., Chen Y., Jia Z., Sun H., Sun Z., Xia X. (2016). Antimicrobial Activity and Possible Mechanism of Action of Citral against *Cronobacter sakazakii*. PLoS ONE.

[6] Yang Z., Xi J., Li J., Qu W. (2009). Biphasic Effect of Citral, a Flavoring and Scenting Agent, on Spatial Learning and Memory in Rats. Pharmacol. Biochem. Behav..

[7] Hajizadeh Moghaddam A., Mashayekhpour M.A., Tabari M.A. (2023). Anxiolytic-like Effects of Citral in the Mouse Elevated plus Maze: Involvement of GABAergic and Serotonergic Transmissions. Naunyn Schmiedebergs Arch. Pharmacol..

[8] Gupta G.L., Samant N.P. (2021). Current Druggable Targets for Therapeutic Control of Alzheimer’s Disease. Contemp. Clin. Trials.

[9] Wojtunik-Kulesza K., Oniszczuk A., Waksmundzka-Hajnos M. (2019). An Attempt to Elucidate the Role of Iron and Zinc Ions in Development of Alzheimer’s and Parkinson’s Diseases. Biomed. Pharmacother..

[10] Bokhoven P., de Wilde A., Vermunt L., Leferink P., Heetveld S., Cummings J., Scheltens P., Vijverberg E. (2021). The Alzheimer’s Disease Drug Development Landscape. Alzheimer’s Res. Ther..

[11] Sedky K., Nazir R., Joshi A., Kaur G., Lippmann S. (2012). Which Psychotropic Medications Induce Hepatotoxicity?. Gen. Hosp. Psychiatry.

[12] Wojtunik K.A., Ciesla L.M., Waksmundzka-Hajnos M. (2014). Model Studies on the Antioxidant Activity of Common Terpenoid Constituents of Essential Oils by Means of the 2,2-Diphenyl-1-Picrylhydrazyl Method. J. Agric. Food Chem..

[13] Wojtunik-Kulesza K.A., Oniszczuk A. (2024). Ability of Selected Monoterpenes to Reduce Fe(III) Ions Being Pro-Neurodegenerative Factors: Tests Based on a FRAP Reaction Extended to 48 Hours. Int. J. Mol. Sci..

[14] Wojtunik-Kulesza K.A., Wiśniewska R. (2022). Interactions of Selected Monoterpenes with Iron and Copper Ions Based on Ferrozine and CUPRAC Methods—The Preliminary Studies. Chem. Biodivers..

[15] Wojtunik-Kulesza K.A., Targowska-Duda K., Klimek K., Ginalska G., Jóźwiak K., Waksmundzka-Hajnos M., Cieśla Ł. (2017). Volatile Terpenoids as Potential Drug Leads in Alzheimer’s Disease. Open Chem..

[16] do Vale T.G., Furtado E.C., Santos J.G., Viana G.S.B. (2002). Central Effects of Citral, Myrcene and Limonene, Constituents of Essential Oil Chemotypes from *Lippia Alba* (Mill.) n.e. Brown. Phytomedicine.

[17] Charret T.S., Pereira M.T.M., Pascoal V.D.B., Lopes-Cendes I., Cristina Rheder Fagundes Pascoal A. (2021). Citral Effects on the Expression Profile of Brain-Derived Neurotrophic Factor and Inflammatory Cytokines in Status Epilepticus-Induced Rats Using the Lithium–Pilocarpine Model. J. Med. Food.

[18] Phillips J.C., Kingsnorth J., Gangolli S.D., Gaunt I.F. (1976). Studies on the Absorption, Distribution and Excretion of Citral in the Rat and Mouse. Food Cosmet. Toxicol..

[19] Marston A., Kissling J., Hostettmann K. (2002). A Rapid TLC Bioautographic Method for the Detection of Acetylcholinesterase and Butyrylcholinesterase Inhibitors in Plants. Phytochem. Anal..

[20] Cetin S., Knez D., Gobec S., Kos J., Pišlar A. (2022). Cell Models for Alzheimer’s and Parkinson’s Disease: At the Interface of Biology and Drug Discovery. Biomed. Pharmacother..

[21] Ruoß M., Vosough M., Königsrainer A., Nadalin S., Wagner S., Sajadian S., Huber D., Heydari Z., Ehnert S., Hengstler J.G. (2020). Towards Improved Hepatocyte Cultures: Progress and Limitations. Food Chem. Toxicol..

[22] van Meerloo J., Kaspers G.J.L., Cloos J. (2011). Cell Sensitivity Assays: The MTT Assay. Methods Mol. Biol..

[23] Bouzenna H., Hfaiedh N., Giroux-Metges M.-A., Elfeki A., Talarmin H. (2017). Biological Properties of Citral and Its Potential Protective Effects against Cytotoxicity Caused by Aspirin in the IEC-6 Cells. Biomed. Pharmacother..

[24] Souza A.C.S., Silva L.K., Queiroz T.B., Marques E.S., Hiruma-Lima C.A., Gaivão I.O.M., Maistro E.L. (2020). Citral Presents Cytotoxic and Genotoxic Effects in Human Cultured Cells. Drug Chem. Toxicol..

[25] Donato M.T., Tolosa L. (2021). High-Content Screening for the Detection of Drug-Induced Oxidative Stress in Liver Cells. Antioxidants.

[26] Uchida N.S., Silva-Filho S.E., Cardia G.F.E., Cremer E., de Souza Silva-Comar F.M., Silva E.L., Bersani-Amado C.A., Cuman R.K.N. (2017). Hepatoprotective Effect of Citral on Acetaminophen-Induced Liver Toxicity in Mice. Evid. -Based Complement. Altern. Med..

[27] Asle-Rousta M., Amini R., Aghazadeh S. (2023). Carvone Suppresses Oxidative Stress and Inflammation in the Liver of Immobilised Rats. Arch. Physiol. Biochem..

[28] Feitosa C.M., de Freitas R.M., Silva V.L., da Silva Araújo L., de Melo C.H.S., Santos F.P.D.S. (2017). Citrus: A Perspective for Developing Phytomedicines for Neurodegenerative Diseases. Citrus Pathology.

[29] Noroozi N., Shayan M., Maleki A., Eslami F., Rahimi N., Zakeri R., Abdolmaleki Z., Dehpour A.R. (2022). Protective Effects of Dapsone on Scopolamine-Induced Memory Impairment in Mice: Involvement of Nitric Oxide Pathway. Dement. Geriatr. Cogn. Disord. Extra.

[30] Giridharan V.V., Thandavarayan R.A., Sato S., Ko K.M., Konishi T. (2011). Prevention of Scopolamine-Induced Memory Deficits by Schisandrin B, an Antioxidant Lignan from Schisandra Chinensis in Mice. Free Radic. Res..

[31] Budzynska B., Boguszewska-Czubara A., Kruk-Slomka M., Skalicka-Wozniak K., Michalak A., Musik I., Biala G. (2015). Effects of Imperatorin on Scopolamine-Induced Cognitive Impairment and Oxidative Stress in Mice. Psychopharmacology.

[32] Opitz B. (2014). Memory Function and the Hippocampus. Front. Neurol. Neurosci..

[33] Beniwal S., Chatterjee I., Gohil N., Ghalami F., Lorenzo-Villegas D., Moradikor N. (2023). Evaluation of the Neuroprotective Activity of Citral Nanoemulsion on Alzheimer’s Disease-Type Dementia in a Preclinical Model: The Assessment of Cognitive and Neurobiochemical Responses. Life Neurosci..

[34] Szutowicz A., Bielarczyk H., Jankowska-Kulawy A., Ronowska A., Pawełczyk T. (2015). Retinoic Acid as a Therapeutic Option in Alzheimer’s Disease: A Focus on Cholinergic Restoration. Expert Rev. Neurother..

[35] Kikonyogo A., Abriola D.P., Dryjanski M., Pietruszko R. (1999). Mechanism of Inhibition of Aldehyde Dehydrogenase by Citral, a Retinoid Antagonist. Eur. J. Biochem..

[36] Gomoll B.P., Kumar A. (2015). Managing Anxiety Associated with Neurodegenerative Disorders. F1000Prime Rep..

[37] Fourtaka K., Christoforides E., Tzamalis P., Bethanis K. (2021). Inclusion of Citral Isomers in Native and Methylated Cyclodextrins: Structural Insights by X-Ray Crystallography and Molecular Dynamics Simulation Analysis. J. Mol. Struct..

[38] Pitucha M., Woś M., Miazga-Karska M., Klimek K., Mirosław B., Pachuta-Stec A., Gładysz A., Ginalska G. (2016). Synthesis, Antibacterial and Antiproliferative Potential of Some New 1-Pyridinecarbonyl-4-Substituted Thiosemicarbazide Derivatives. Med. Chem. Res..

[39] Venault P., Chapouthier G., de Carvalho L.P., Simiand J., Morre M., Dodd R.H., Rossier J. (1986). Benzodiazepine Impairs and β-Carboline Enhances Performance in Learning and Memory Tasks. Nature.

[40] Allami N., Javadi-Paydar M., Rayatnia F., Sehhat K., Rahimian R., Norouzi A., Dehpour A.R. (2011). Suppression of Nitric Oxide Synthesis by L-NAME Reverses the Beneficial Effects of Pioglitazone on Scopolamine-Induced Memory Impairment in Mice. Eur. J. Pharmacol..

[41] Skalicka-Wozniak K., Budzynska B., Biala G., Boguszewska-Czubara A. (2018). Scopolamine-Induced Memory Impairment Is Alleviated by Xanthotoxin: Role of Acetylcholinesterase and Oxidative Stress Processes. ACS Chem. Neurosci..

[42] Yan F., Robert M., Li Y. (2017). Statistical Methods and Common Problems in Medical or Biomedical Science Research. Int. J. Physiol. Pathophysiol. Pharmacol..

